# Does the Fissure Last Technique Really Reduce Postoperative Airleak After Lung Resection? Results From a Prospective Randomized Controlled Trial

**DOI:** 10.1093/icvts/ivag039

**Published:** 2026-02-13

**Authors:** Federico Vaisitti, Simona Sobrero, Stefano Rudella, Alessandra Russo, Marco Marcaccini, Luca Errico, Francesco Leo

**Affiliations:** Thoracic Surgery Division, Oncology Department, S. Luigi Gonzaga Hospital, University of Turin, Orbassano, Turin 10043, Italy; Thoracic Surgery Division, Oncology Department, S. Luigi Gonzaga Hospital, University of Turin, Orbassano, Turin 10043, Italy; Thoracic Surgery Division, Oncology Department, S. Luigi Gonzaga Hospital, University of Turin, Orbassano, Turin 10043, Italy; Thoracic Surgery Division, Oncology Department, S. Luigi Gonzaga Hospital, University of Turin, Orbassano, Turin 10043, Italy; Thoracic Surgery Division, Oncology Department, S. Luigi Gonzaga Hospital, University of Turin, Orbassano, Turin 10043, Italy; Thoracic Surgery Division, Oncology Department, S. Luigi Gonzaga Hospital, University of Turin, Orbassano, Turin 10043, Italy; Thoracic Surgery Division, Oncology Department, S. Luigi Gonzaga Hospital, University of Turin, Orbassano, Turin 10043, Italy

**Keywords:** lung resection, prolonged airleak, fissure first, fissureless, postoperative complications

## Abstract

**Objectives:**

The fissure last (FL) technique described in 1998 has the goal of minimizing postoperative airleak leaving the stapling of the fissure as the last step. The aim of this study was the reduction in airleak may be already evident 48 hours after surgery.

**Methods:**

The hypothesis of the study was that the routine of adopting the FL technique during lung resection for NSCLC active may reduce the number of patients presenting an airleak 48 hours after surgery assessed by a digital device. The study was designed as a prospective phase III single-centre 1:1 randomized trial, which compared the FL technique with the standard fissure first (FF) technique. Sample size was calculated assuming a 50% reduction of airleak at 48 hours in the FL group, and it was set at 150 cases according to previously published evidence.

**Results:**

Regarding the main end-point, air leaks on POD2 were 5% lower in the FL group as compared to the FF group (50% vs 55%, *P* .51) and that difference was maintained on POD 5 (26% vs 32%, *P* .42). In the subgroup of patients in which a higher rate of airleak was expected (Walker 3 and 4, *n* = 33), the adoption of the FL technique did not show a significant benefit in terms of prolonged airleak (PAL), chest drain duration and hospital stay.

**Conclusions:**

Results from this study suggest that the FL strategy does not confer any benefit in terms of PAL, even in the context of an incomplete fissure. Clinical registration number: n 184/2020, 5/11/2020, EudraCT2020-004900-33

## Graphical abstract

**Figure:**
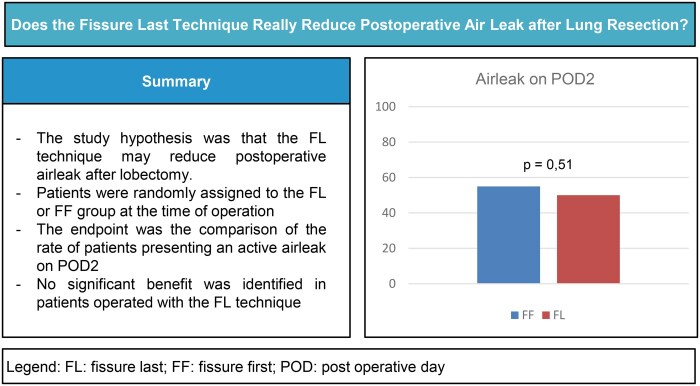

## INTRODUCTION

Prolonged airleak (PAL) often affects postoperative outcome after lung resection, delaying drain removal and hospital discharge.^1–3^ The leak is usually originating deeply in the fissure, where hilar structures are isolated before completing lobe separation. There are alternatives to this technical sequence, one of which is intended to maximally reduce fissure injury, the fissure last, called also fissureless technique (FL). This strategy, described in 1998, has the goal of minimizing lung injury by isolating vascular and bronchial structures first, leaving the stapling of the fissure as the last step.^4^

Since its first description, the fissure last (FL) technique has been widely adopted as it conjugates well, especially with the ergonomics of minimally invasive techniques such as Vats lobectomy, which gained a central position in the management of early-stage lung cancer.^5^ In a randomized trial comparing FL to the standard fissure first technique (FF), the incidence of PAL was reduced from 33% to 3%.^6^ A meta-analysis published in 2017, collecting more than 800 cases, suggested that the FL technique may reduce PAL by 60%.^7^

Despite a strong rationale, the FL technique did not reduce postoperative PAL in clinical practice over time, and the reason for that is probably due to its multifactorial nature. In fact, apart from surgical technique, many other elements may contribute to the occurrence of PAL, such as pleural adhesions, completeness of the fissure, use of sealants,^8^ and intraoperative management of bubbles at the end of the operation,^9^^,^^10^ all elements which are carefully analysed only in the setting of a controlled trial. Another potential confounding variable and source of bias is that nowadays the concept of enhanced recovery after surgery (ERAS^11^) made the classical definition of prolonged airleak (lasting 5 days or more) probably obsolete and inaccurate, as many patients are already at home at POD5.

The aim of this study was to verify the potential benefits of the FL technique on early discharge after lung resection in a randomized trial, testing the hypothesis that the benefit of the FL technique may be already evident 48 hours after surgery.

## METHODS

The hypothesis of the study was that the routine of adopting the fissure last technique during lung resection for NSCLC active may reduce the number of patients presenting an airleak 48 hours after surgery assessed by a digital device. The main end-point of the study was the comparison between groups in terms of the presence of airleak on POD2 as a binary phenomenon (no = 0 mL/minute, yes >0 mL/minute). Secondary end-points were the assessment of the pleural residual space and the analysis of other factors potentially relevant in determining postoperative airleak.

The study was designed as a prospective phase III single-centre 1:1 randomized trial that compared the FL technique with the standard fissure first (FF) technique. Sample size was calculated according to existing data at the time of study design, mainly a randomized trial showing a 10 times lower PAL in patients treated with FL technique and a meta-analysis suggesting a RR of 0.4 for FL technique.^12^ Therefore, assuming a 50% reduction of air leak at 48 hours in the FL group, a margin error of 5%, and a confidence interval of 95%, the sample size was set at 150 cases. The protocol (3 F Trial) received approval by the local ethical Committee (n 184/2020, 5/11/2020) and was then registered (EudraCT CT2020-004900–33). Collection and storage of data from included patients is consistent with requirements outlined in the WMA Declaration of Taipei. Each patient has provided written informed consent. Candidates for lobectomy for the treatment of NSCLC were considered eligible if aged 18 or more and fit for general anaesthesia. Exclusion criteria were prior ipsilateral surgery or planned resection other than lobectomy (segmentectomy, bilobectomy, sleeve lobectomy, or pneumonectomy) or cases performed by residents. Every case was discussed by the institutional tumour board before the surgical decision.

Randomization was performed using a computer-generated block sequence (block size 6) on the day of surgery and was revealed to the surgeon at the beginning of the procedure. A minimally invasive approach (Vats or Rats) was used, except for large tumors (≥7 cm in diameter), bulky N1 disease, and multiple non-bulky N2 disease. At the beginning of the procedure, pleural adhesions, when present, were classed according to Li’s classification^13^ and the fissure was classed according to Walker’s classification.^14^ In FL cases, fissure development and lobe separation were obtained by stapling (Signia, Medtronic for Vats cases and Sureform, Intuitive for Rats cases) after vascular and bronchial division. In FF cases, the fissure was completed before vascular and bronchial section using the same type of staplers as in the FL group, unless in the case of a complete fissure. Systematic nodal dissection was performed during the operation. At the end of the procedure, the presence of intraoperative air leaks was assessed and managed according to the surgeon’s preference (suturing and/or sealants).

**Figure 1.**
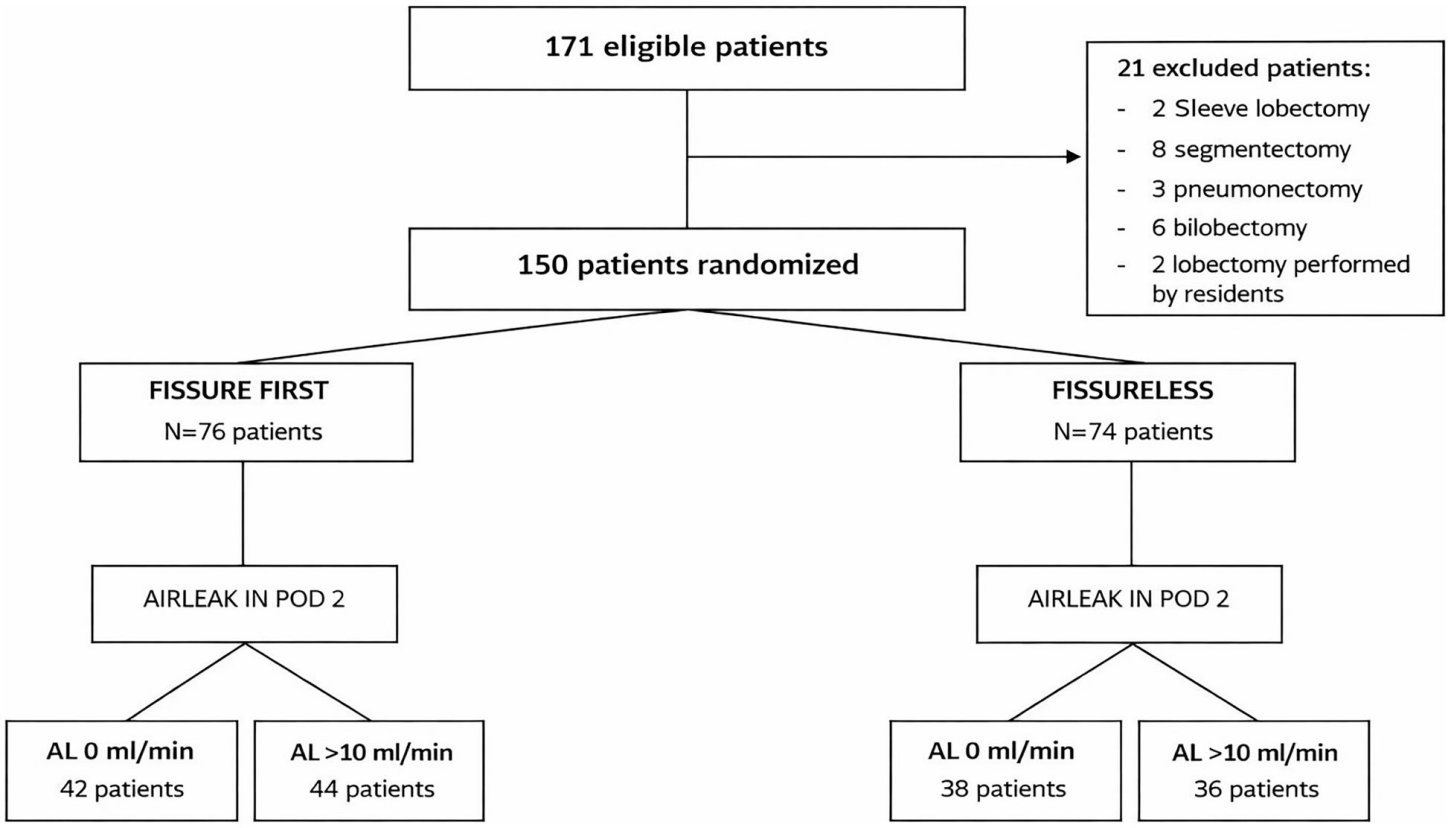
Flowchart. Postoperative management according to the presence of airleak: chest drain removal and radiological assessment. Abbreviations CXR, chest X-ray; Pnx, pneumothorax; POD, postoperative day.

After surgery, a 28 Ch chest drain was left in the pleural cavity and connected to a digital device (Thopaz+, Medela, Suisse) under continuous suction at 15cmH_2_O. Mobilization started in the morning of POD1 when possible, and a chest X-ray was performed on POD1 and POD2. The presence of airleak was checked in the morning of POD2, and the drain removed when airleaks were absent. In case of airleak ≤ to 50 mL/minute, the drain was removed after 24 hours of negative clamping test. In case of airleak > to 50 mL/minute, the patient was connected to a Heimlich valve and discharged when feasible (**Figure 1**). Patients were contacted by phone on 3 days after discharge and weekly after; meanwhile, on-site follow-up visits were scheduled 15 and 30 days after discharge.

Residual pleural space was investigated on the POD2 chest X-ray by a senior radiologist unaware of the randomisation group and classed according to Collins’s method.^15^ The size of pneumothorax was measured at the apex (A), at the midpoint of the upper zone (B) and at the midpoint of the lower zone (C), and the percentage of the pneumothorax was calculated according to the formula 4.2 + 4.7(A + B+C). Subgroup analysis was conducted according to the type of surgical access (open vs minimally invasive), presence of adhesions, and fissure classification according to Walker descriptors. Other factors potentially relevant for airleak occurrence (such as smoking history, COPD, use of sealants) were analysed by univariable analysis and inserted into a multivariable analysis when reaching a level of significance (*P*-value < .05). The main end-point was assessed by an intention-to-treat analysis. Continuous variables were compared using Student’s *t*-test or Mann-Whitney test as appropriate. Categorical variables were analysed by Chi-squared test. Statistical analysis was performed by SPSS version 29.

## RESULTS

**Figure 2.**
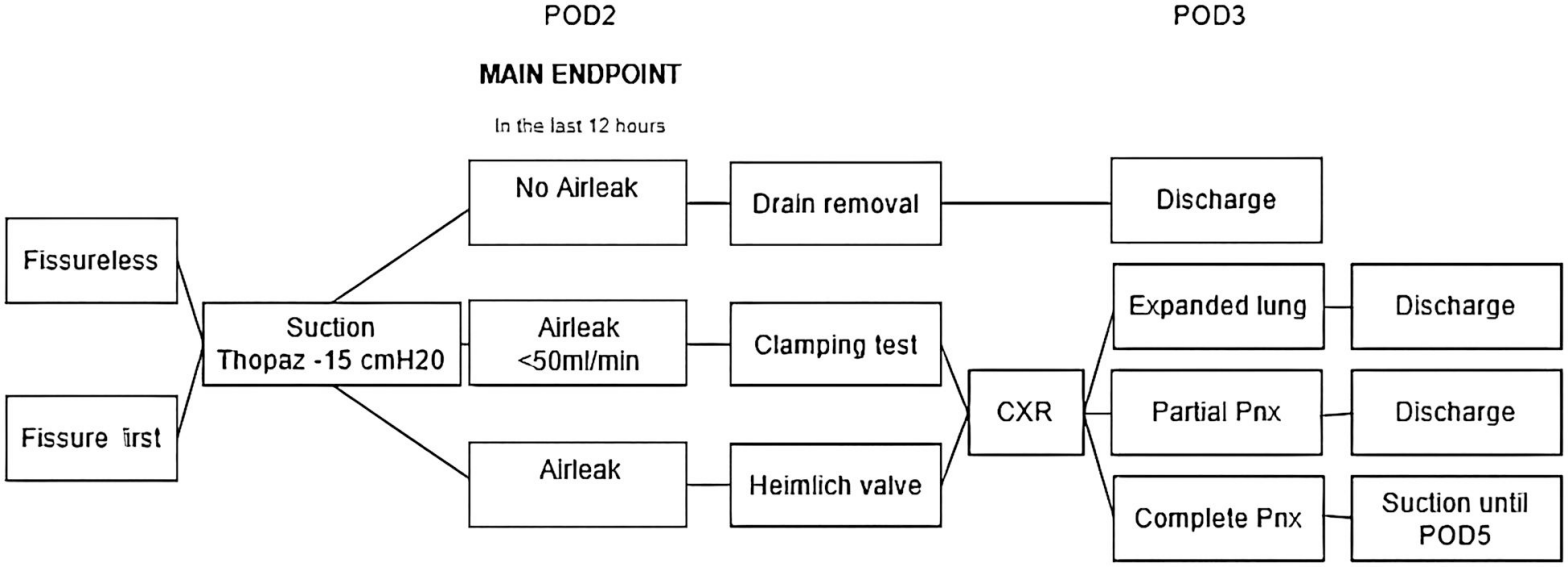
Flowchart Illustrating the Study Population and 1:1 Randomization into the Fissure-First and Fissureless Groups. Patient and chest tube management according to the presence of postoperative airleak. Abbreviations: AL, airleak; POD, post operative day.

One hundred and fifty patients were enrolled from January 2021 to June 2023, 74 in the FL group and 76 in the FF group (**Figure 2**). Clinical characteristics are listed in **Table 1**. Intraoperative findings (**Table 2**) modified the surgical strategy in 2 cases in the FL group (1 sleeve lobectomy and 1 unexpected pleural dissemination) and in one case in the FF group (sleeve lobectomy). On POD2, an active airleak was detected in 79 patients (47%, **Table 3**), more frequently in females and patients with complete fissures.

**Table 1.** Baseline Patient Characteristics

Variables	**Fissure first** **(*n*: 76; 51%)**	**Fissureless** **(*n*: 74; 49%)**	*P*-value
Sex			
Male	36 (46%)	49 (67%)	.01
Female	40 (54%)	25 (33%)	
Age, average (SD)	70.3 (7.9)	69 (8.6)	.40
Smoking history			
Yes	57 (76%)	60 (82%)	.43
No	19 (24%)	14 (18%)	
Comorbidities			
COPD	16 (21%)	14 (19%)	.10
NIDDM	10 (13%)	7 (9%)	.51
FEV1			
Lt/min (SD)	2.2 (0.7)	2.5 (0.8)	.45
% (SD)	94.2 (23.7)	99.4 (20.7)	.07
DLCO % (SD)	78.5 (17.1)	83.8 (18)	.03
cTNM			
I-IIA	61 (80%)	54 (73%)	1.14
>IIB	15 (20%)	20 (27%)	
ASA score			
<2	7 (9%)	9 (12%)	.34
>3	69 (91%)	65 (88%)	

**Abbreviations:** COPD: chronic obstructive pulmonary disease; DLCO, diffusing capacity for carbon monoxide; FEV1, forced expiratory volume in the first second; NIDDM, non-insulin dependent diabetes mellitus; SD, standard deviation.

**Table 2.** Descriptive Intraoperative Variables

Variables	**Fissure first** **(*n*: 76; 51%)**	**Fissureless** **(*n*: 74; 49%)**	*P*-value
Type of lobectomy			
Upper lobe	39 (51)	35 (47)	.24
Lower lobe	37 (49)	39 (53)	
Surgical approach			
Open	21 (27)	26(35)	.98
Minimally Invasive	55 (73)	48 (65)	
Duration of surgery (minutes)	251	243	.39
Stapler (*n*, median)	4	4	.60
Sealant			
Yes	49 (64)	38 (51)	.10
No	27 (36)	36 (49)	
Fissure according to Walker’s classification			
1-2	58 (76)	59 (79)	.61
3-4	18 (24)	15 (21)	
Score adhesion according to Li’s classification			
I-II	72 (95)	70 (95)	.96
III-V	4 (5)	4 (5)	

**Table 3.** Descriptive Patient Characteristics With/Without Airleak on POD 2

Variables	**No airleak** **(*n* 71; 53%)**	**Airleak** **(*n* 79; 47%)**	*P*-value
Sex			
Male	47 (66)	38 (48)	.02
Female	24 (34)	41 (52)	
Age, average (SD)	69.5 ± 8.2	70.7 ± 8.3	.18
FEV1			
Lt/min (average)	2.35 (0.4)	2.36 (0.2)	.22
DLCO % (average)	81.5 (1.1)	79.4 (0.8)	.36
Fissure approach			
Fissure first	34 (48)	42 (53)	.51
Fissureless	37 (52)	37 (47)	
Fissure according to Walker’s classification			
1-2	49 (69)	67 (72)	.02
3-4	22 (31)	12 (28)	
Pleural sealant			
Yes	46 (65)	39(49)	.05
No	25 (35)	40 (51)	
Chest tube removal day (median)	3	7	.01
Hospital stay (day, median)	4	7	0.01

Abbreviations: DLCO, diffusing capacity for carbon monoxide; FEV1, forced expiratory volume in the first second; SD, standard deviation.

Regarding the main end-point, airleaks on POD2 were 5% lower in the FL group as compared to the FF group (50% vs 55%, *P* .51) and that difference was maintained on POD 5 (26% vs 32%, *P* .42). Groups were also equivalent in terms of duration of drain (median of 4 days in each group, *P* .77) and hospital stay (5 days, *P* .71). The rate of patients discharged on Heimlich valve was 9% lower after FL (16% vs 25%, *P* .18) (**Table 4**).

**Table 4.** Primary Outcomes

Variables	**Fissure first** **(*n*: 76; 51%)**	**Fissureless** **(*n*: 74; 49%)**	*P*-value
Airleak on POD2			
No	34 (45)	37 (50)	.51
Yes	42 (55)	37 (50)	
Chest X-ray on POD2			
Apical pneumothorax	20 (26)	14 (19)	.27
Expanded	56 (74)	60 (81)	
Collins index % (SD)	17.5 (7.1)	14.3 (7)	.11
Chest tube removal day (median)	4	4	.77
PAL > POD 5			
Yes	24 (32)	19 (26)	.42
No	52 (68)	55 (74)	
Discharge with Heimlich valve			
Yes	19 (25)	12 (16)	.18
No	57 (75)	62 (84)	
Hospital stay (day, median)	5	5	.71
Chest X-ray on POD 30			
Apical pneumothorax	11 (15)	8 (12)	.50
Expanded	65 (85)	66 (88)	
Collins index % (SD)	6.5 (1.8)	5.4 (2.1)	.24

Abbreviations: PAL, prolonged airleak; POD, postoperative day.

A complete lung re-expansion was recorded at chest X-ray on POD2 in 81% of patients in the FL group and in 74% of patients in the FF group (*P* .27). The size of detected pneumothorax was 14.3% in the FL group and 17.5% in the FL group (*P* .11). This difference did not affect the duration of chest drain in both groups.

In the subgroup of patients in which a higher rate of airleak was expected (Walker 3 and 4, *n* = 33), the adoption of the FL technique did not show a significant benefit in terms of PAL, chest drain duration, and hospital stay. In FL patients, lung expansion was complete in a reduced number of cases as compared to FF patients (53% vs 75%, *P* .02), without any difference in terms of hospital stay and drain duration (**Table 5**).

**Table 5.** Patients With 3–4 Fissure

Variables	**Fissure-first** **(*n* = 18)**	**Fissureless** **(*n* = 15)**	*P*-value
Airleak on POD 2			
No	8 (44)	5(33)	.51
Yes	10 (56)	10(67)	
Chest X-ray on POD 2			
Not expanded	2 (25)	7 (47)	.02
Expanded	16 (75)	8 (53)	
Collins index % (SD)	16.5 (1.5)	9.4 (2.9)	NV
POD chest tube removal (day, median)	8	7	.34
PAL > POD 5			
Yes	8 (44)	7 (47)	.89
No	10 (56)	8 (53)	
Discharged with Heimlich valve			
Yes	8 (44)	5 (33)	.51
No	10 (56)	10 (67)	
Chest X-ray on POD 30			
Not expanded	4 (29)	3 (20)	.87
Expanded	14 (71)	12 (80)	
Collins index % (SD)	6 (1.2)	6.6 (2.3)	NV

Abbreviations: PAL, prolonged airleak; POD, post-operative day; SD: standard deviation.

## DISCUSSION

Unexpectedly, results from our study did not confirm that the fissure-last strategy reduces the incidence of postoperative airleak after lobectomy when adopted routinely. This information is counterintuitive but fits with the real-world observation that, despite the wide adoption of FL lobectomy performed by minimally invasive techniques, the occurrence of postoperative airleak still remains a clinical problem prolonging hospital stay and chest drain duration.^16^

Several aspects of this study may potentially dilute the benefits of the FL technique. First of all, in this study, the main end-point (PAL) was measured on POD2, while the standard measure of PAL is taken on POD5. The reason for this choice is that nowadays many patients are already at home 5 days after surgery. As a consequence, PAL measured on POD5 is a granular and inaccurate marker, as when the drain is removed after discharge, airleak often stops several days before. On the contrary, measuring PAL on POD2 is in line with Eras principles, accurate, and from the statistical point of view, detects more events than on POD5, therefore a dilution effect is unlikely.

Another issue is the technique used for the detection of airleak. The traditional assessment by the water seal valve generates a relevant inter-operator variability. In this trial, the measure was standardized by the use of a digital device, reducing that variability. A peculiar aspect of the study was also the restrictive threshold defining the absence of airleak (<10 mL/minute). That value was decided according to our previous experience^17^ in order to maximally reduce the risk of pneumothorax after drain removal and the consequent increased respiratory workload. This is also the reason why, in doubtful cases (10–50 mL/minute), a clamping test was adopted instead of removing the drain directly. This strategy considers equally important early discharge and residual pleural space, and it may be questionable, but it affected both groups, and a bias effect is unlikely.

The most questionable issue remains the routine use of suction after lobectomy, as a potential detrimental effect has been reported by some studies.^17^^,^^18^ The adoption of suction reflects our standard practice, justified by 2 reasons: (1) a large residual pleural space is not considered an acceptable result and (2) suction may stratify patients according to the type of airleak, which is not a binary phenomenon. In our experience, suction allows to define whether the airleak is laminar or turbulent simply by increasing suction and assessing the airleak (the “lung-stress test,” unpublished data): according to the Poiseuille law, if the increase in airleak is less then proportional to the suction increase, the flow is turbulent, lung expansion is easier and the leak lasts shorter meanwhile if the airleak increase is proportional to the increase in suction, the leak is laminar, the risk of large pneumothorax after drain removal is doubled and the airleak lasts longer.

The final question is: was the study underpowered? The study hypothesis was set with an expected PAL reduction of 50% on POD2. This decision was based on results from a previous randomized trial,^12^ suggesting that airleak is 10 times lower after FL lobectomy. Additionally, a meta-analysis on more than 800 cases suggested a RR of 0.4 for the FL technique.^7^ Additionally, in the study design, the main end-point was measured on POD2 by a digital device instead of POD5, further magnifying the expected benefit. Finally, despite the limited dimension of the cohort, the single-centre design of the study limited variability due to different intraoperative airleak management, potentially introducing hardly identifiable bias.

The reason explaining the lack of a relevant benefit of FL lobectomy is probably multifactorial. On one side, there is a technical aspect: at the end of the FL lobectomy, traction is applied to the bronchial stump. The area is then dissected to create the space for stapling and that manoeuvre may create a potential source of airleak. On the other side, in the study, a conservative attitude regarding the residual pleural space was adopted in order to reduce the incidence of large pneumothorax after drain removal. It is possible that a drain management strategy less attentive to residual pleural space would have highlighted a stronger difference between groups. Even more important, the lack of benefits may be attributable to the heterogeneity of Walker 3-4 patients between different studies.

## CONCLUSIONS

In conclusion, results from this study suggest that the FL strategy does not confer any benefit in terms of PAL, even in the context of an incomplete fissure. Nevertheless, larger randomized trials are needed to measure the impact of a single technical factor in a multifactorial event as PAL is difficult.

## Data Availability

The data underlying this article will be shared on reasonable request to the corresponding author.
